# Ultra-sparse Connectivity within the Lateral Hypothalamus

**DOI:** 10.1016/j.cub.2020.07.061

**Published:** 2020-10-19

**Authors:** Denis Burdakov, Mahesh M. Karnani

**Affiliations:** 1 Laboratory of Neurobehavioral Dynamics, Institute for Neuroscience, Department of Health Sciences and Technology, ETH Zürich, Zürich 8603, Switzerland; 2 The Francis Crick Institute, London NW1 1AT, UK; 3 Institute of Psychiatry, Psychology & Neuroscience, King’s College London, London SE5 8AF, UK; 4 Neuroscience Center Zürich (ZNZ), ETH Zürich and University of Zürich, Zürich 8057, Switzerland; 5 Université de Paris, Saints-Pères Paris Institute for the Neurosciences (SPPIN), CNRS, Paris 75006, France

**Keywords:** lateral hypothalamus, synaptic connectivity, patch clamp, orexin, MCH, gamma oscillation

## Abstract

The lateral hypothalamic area (LH) is a vital controller of arousal, feeding, and metabolism [1, 2], which integrates external and internal sensory information. Whereas sensory and whole-body output properties of LH cell populations have received much interest, their intrinsic synaptic organization has remained largely unstudied. Local inhibitory and excitatory connections could help integrate and filter sensory information and mutually inhibitory connections [3] could allow coordinating activity between LH cell types, some of which have mutually exclusive behavioral effects, such as LH VGLUT2 and VGAT neurons [4, 5, 6, 7] and orexin- (ORX) and melanin-concentrating hormone (MCH) neurons [8, 9, 10]. However, classical Golgi staining studies did not find interneurons with locally ramifying axons in the LH [11, 12], and nearby subthalamic and thalamic areas lack local synaptic connectivity [13, 14]. Studies with optogenetic circuit mapping within the LH have demonstrated only a minority of connections when a large pool of presynaptic neurons was activated [15, 16, 17, 18, 19]. Because multiple patch clamp has not been used to study LH connectivity, aside from a limited dataset of MCH neurons where no connections were discovered [15], we used quadruple whole-cell recordings to screen connectivity within the LH with standard methodology we previously used in the neocortex [20, 21, 22]. Finding a lack of local connectivity, we used optogenetic circuit mapping to study the strength of LH optogenetic responses and network oscillations, which were consistent with ultra-sparse intrinsic connectivity within the LH. These results suggest that input from other brain structures is decisive for selecting active populations in the LH.

## Highlights

• LH neurons within <300 μm are almost never connected synaptically
• Local optogenetic responses are consistent with ultra-sparse connectivity
• Local beta-gamma oscillations cannot be driven optogenetically in the LH

## Graphical Abstract

**Figure:**
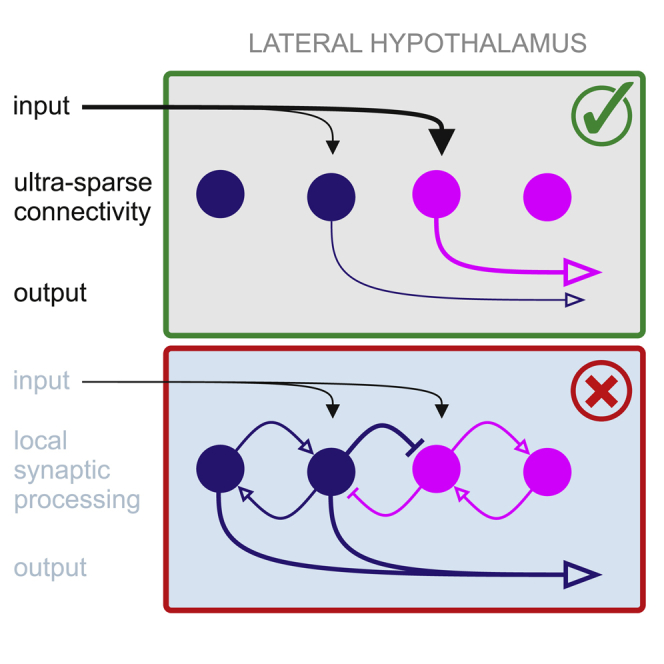

## Teaser

It is unknown how neural activity is coordinated among lateral hypothalamic area (LH) neurons, which generate fundamental behavioral actions such as attack and evasion. Burdakov and Karnani show there is near-zero local connectivity in the LH, suggesting that incoming synaptic input is integrated primarily within individual neurons.

## Results

### Ultra-sparse Connectivity with Multiple Patch-Clamp Recordings

**Figure 1:**
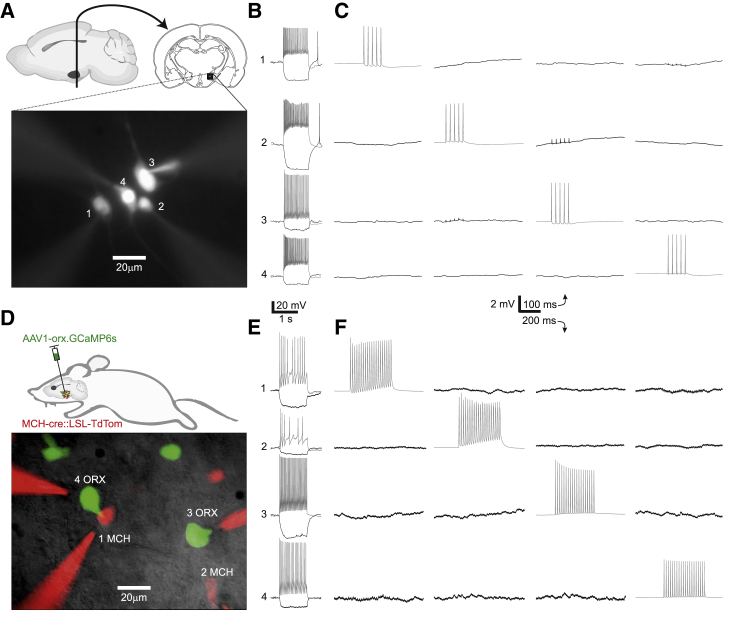
Example Quadruple Whole-Cell Recordings from LH Slices (A) Schematic of recording location and fluorescence micrograph of a recording configuration of four unidentified (n.m.) neurons in the LH. (B) Membrane potential and firing responses of neurons shown in (A) to a −30-pA (black) and 150-pA (gray) current injection. Cells were held at −50 mV at baseline. (C) Example connectivity recording of cells in (A) and (B), with tested presynaptic cells firing 5 action potentials at 50 Hz (gray) and tested postsynaptic cells showing lack of synaptic responses (black). Traces are averages of 50 trials. (D) Schematic of the labeling strategy for identifying orexin and MCH neurons using three transgenes, and fluorescence micrograph of an example recording configuration with two MCH cells and two orexin cells. (E) Neurons in (D) recorded as in (B). (F) Neurons in (D) and (E) recorded as in (C), except with a longer train of action potentials. Traces are averages of 30 trials. Scale bars below (B) and (C) apply to (E) and (F), respectively. The vertical scale in (C) and (F) only applies for black traces.

To begin looking for synaptic connectivity within the lateral hypothalamic area (LH), we used standard methodology to cut brain slices and obtain multiple simultaneous whole-cell patch-clamp recordings in wild-type animals (Figures 1A and 1B). We sequentially imposed a 50-Hz train of 5 action potentials on each recorded neuron while monitoring membrane potential fluctuations in the other neurons. Although this approach has revealed synaptic connectivity in the neocortex [21, 22], we found no connections in the LH (0 connected in 248 tested putative connections; Figure 1C).

**Figure 2:**
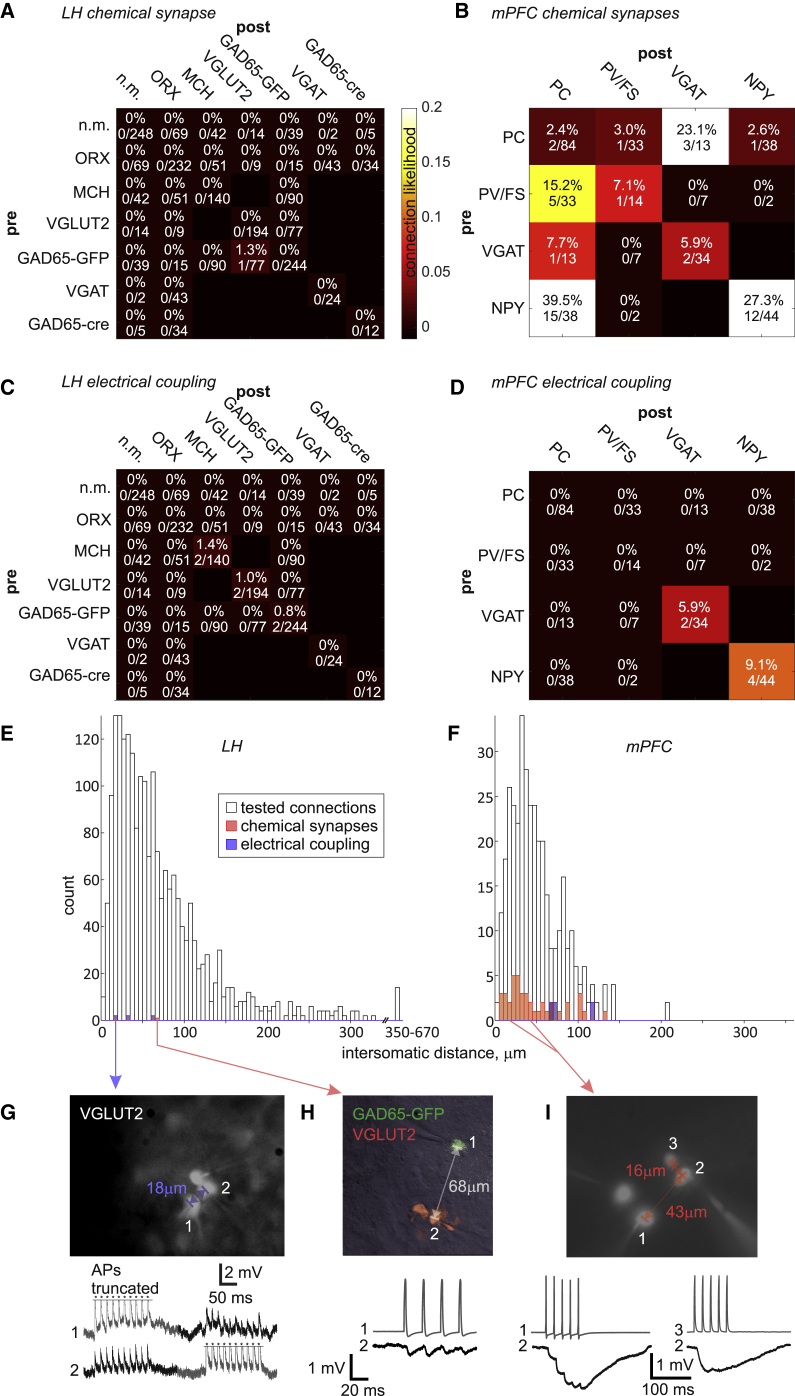
Analysis of Connectivity Recordings in the LH and mPFC (A) Overall synaptic connectivity within the LH across 2,074 tested connections, showing 1 synapse. (B) Overall synaptic connectivity within the mPFC across 362 tested connections, showing 43 synapses. (C) Electrical coupling in the LH was found in 3 cell pairs among 1,037 tested. (D) Electrical coupling in the mPFC was found in 3 cell pairs among 181 tested. (E and F) Distances between cell somata in tested connections in the LH (E) and mPFC (F). (G–I) Example micrographs and voltage recordings of identified connections. (G) Action potentials (APs) are truncated, as indicated by asterisks. (H) The only local chemical synapse found within the LH. (I) An example recording from the mPFC with an NPY neuron (3) and a PV/FS neuron (1) inhibiting a pyramidal cell (2). Vertical scale bars in (H) and (I) apply only to postsynaptic traces (black).

Because it is possible that connectivity is specific to neuronal subtypes, we used multiple transgenes to identify genetically defined LH subpopulations in acute slices through fluorescent protein expression. This approach (STAR Methods) allowed us to target up to two identified populations (e.g., orexin- [ORX] and melanin-concentrating hormone [MCH] neurons labeled in Figures 1D and 1E) as well as the non-marked (n.m.) neurons outside these populations. With this approach, we also found zero connectivity in all but one category (1/77 tested GAD65-GFP→VGLUT2 putative connections was connected) between and within the cardinal LH populations orexin, MCH, VGLUT2, GAD65-GFP, GAD65-cre, VGAT-cre, and n.m. neurons [2, 23]. Overall, 1 synaptic connection was found among 2,074 tested (Figures 1F and 2A).

We took several measures to control for potential sources of artifact in this important result. We verified that slice geometry is not a confounding factor by testing connections in horizontal (0 connected in 74 tested), sagittal (0 connected in 50 tested), and coronal slices (1 connected in 1,950 tested). Because the distance from the slice surface can conceivably affect connectivity through limiting the available space where synapses might have existed in the intact brain, we monitored the depths of recorded neurons (37.2 ± 22.5 μm, range 12–123 μm, n = 50). These depths are within the reported range (5–130 μm) of reliable connectivity measurements reported before [24]. Because preservation of connections can vary across individual slices, we report that in the slice with the one recorded synapse, 17 other trials revealed no connections, and in the adjacent slices from that animal, 36 other trials revealed no connections. Because the age of animals can affect connectivity, we report the age of this animal was post-natal day 45 (P45), which is far from the low end of distribution of ages in this study (P21–P201, mean P94.8 ± 42.3). Finally, to show that nothing in the methodology systematically affects synaptic connectivity, we recorded with the same equipment and preparation protocol a control dataset from the medial prefrontal cortex (mPFC; including infralimbic, prelimbic, and anterior cingulate cortices). In these control data, 43 synaptic connections were observed out of 362 trials (Figure 2B). These connections were more frequent among inhibitory neurons, as has been shown by many other studies [25, 26].

Bidirectional electrical coupling among LH neurons was observed on three occasions, once each among pairs of MCH, GAD65-GFP, and VGLUT2 neurons (Figure 2C). This overall electrical coupling rate of 0.3% (6/2,074) was contrasted by a rate of 1.7% (6/362) in the mPFC (Figure 2D). In both brain structures, this very sparse electrical coupling was found within genetically defined subpopulations, which is typical in the cortex [25]. The electrical coupling coefficient (STAR Methods) was 0.075 ± 0.102 in the LH and 0.018 ± 0.013 in the mPFC (p = 0.2), values which are typical for the adult CNS [27].

Because synaptic connectivity is known to be distance dependent, we measured intersomatic distances from all experiments (Figures 2E and 2F). Distances in the LH (mean 71.6 ± 65.7 μm) were significantly higher than distances in the mPFC (mean 50.3 ± 32.4 μm, p < 10^−6^ by Wilcoxon rank-sum test) because, failing to find connections at short distances, we searched for possible longer-distance connections. The range of covered distances was broader in the LH (3.0–664.5 μm) than mPFC (4.4–208.7 μm), and encompassed all mPFC distance bins and exceeded them in number (Figures 2E and 2F). The intersomatic distance bins of the discovered synapses were well sampled, indicating that the measured connectivity rates are reliable. The discovered synapse in the LH was inhibitory and weaker than typical synapses in the mPFC (Figures 2G–2I). We therefore conclude that local synaptic connectivity in the LH, benchmarked to that in the neocortex, is nearly non-existent.

### Single-Synapse Equivalent Connections with Optogenetic Population Activation

**Figure 3:**
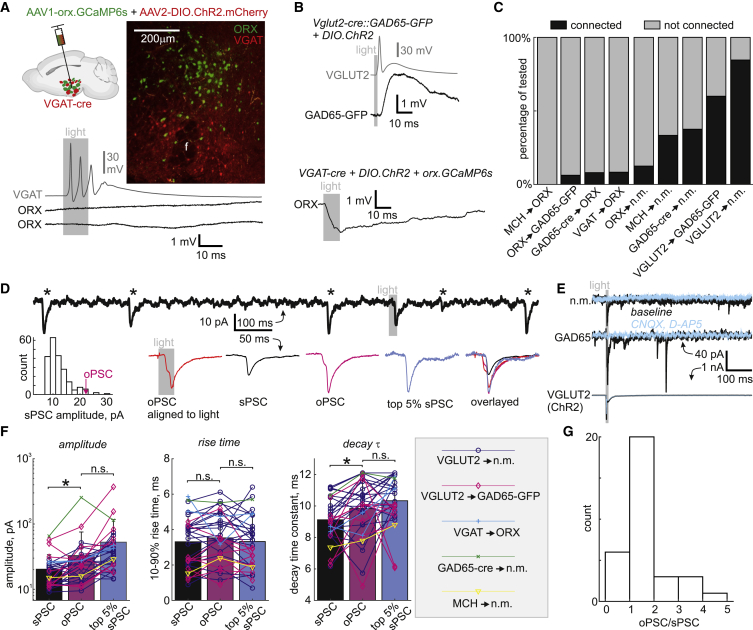
Optogenetic Connections within the LH Are Consistent with Ultra-sparse Synaptic Connectivity (A) Top: schematic of opsin and label delivery strategy to identify connections from VGAT neurons to orexin neurons. Middle: micrograph of an example coronal section with VGAT neurons expressing ChR2.mCherry and orexin neurons expressing GCaMP6s. f, fornix. Bottom: current-clamp recording shows a typical lack of synaptic response in orexin neurons while the simultaneously recorded VGAT neuron spikes in response to the light stimulus. (B) Examples of identified connections from the optogenetically activated VGLUT2 population to a GAD65-GFP neuron (top) and from the VGAT population to an orexin neuron (bottom). Amplitude scale bars in (A) and (B) only apply to black traces. Gray traces illustrate spiking in the channelrhodopsin-bearing population. (C) Summary data from all recordings showing that overall only 32% (43/136) of recorded neurons had a postsynaptic response when thousands of presynaptic neurons were activated. (D) Example voltage-clamp (−60 mV) recording of oPSCs and sPSCs from the GAD65-GFP neuron shown in (B), when VGLUT2 neurons were optogenetically tagged. Top: the trace is a segment of the recording showing a light-induced PSC and several sPSCs (asterisks). Bottom: from left, histogram of sPSC amplitudes and the average oPSC amplitude labeled, average oPSC across all light stimuli, average sPSC, average oPSC (detected and aligned with the same method as the sPSC for a reliable comparison), average of the largest 5% of sPSCs, and the average waveforms overlaid. (E) Another example recording of an n.m., GAD65-GFP, and VGLUT2-cre::DIO.ChR2 neuron simultaneously before (baseline, black) and after glutamate receptor blockade (CNQX, D-AP5, light blue). The VGLUT2 neuron shows runaway action currents indicating reliable spiking in response to light. (F) Summary data of average PSCs as labeled in (D) across all voltage-clamp data, showing that oPSCs were larger than sPSCs but not significantly different from the top 5% of sPSCs. ^∗^p < 0.05; n.s., p > 0.05. The y axis in the amplitude plot is logarithmic. Error bars denote SD. (G) Histogram of the oPSC/sPSC amplitude ratio for all neurons in (F), showing the majority of optogenetic connections can be explained by 1 or 2 sPSCs.

Having found a surprising lack of synaptic connectivity with multiple whole-cell recordings, we sought to reconcile our result with previously demonstrated connections using optogenetics [15, 16, 17, 18]. We first assessed several population-specific connections optogenetically, confirming in each case that the optogenetically labeled presynaptic population was firing action potentials in response to light (Figures 3A–3C; all tested cells depolarized and spiking was induced in 7/11 VGAT, 16/20 ORX, 13/19 GAD65, 14/16 VGLUT2, and 3/3 MCH cells). Connections were overall rarer than “non-connections” (43/136; Figure 3C), and the postsynaptic response amplitudes were low considering they arise from the firing of thousands of presynaptic neurons (MCH→ORX 0/5; ORX→GAD65-GFP 1/16, 0.3 mV; GAD65-cre→ORX 2/25, 2.0 and 3.1 mV; VGAT→ORX, 2/24, 0.6 and 0.6 mV; ORX→n.m. 1/8, 0.1 mV; MCH→n.m. 3/9, 0.3 ± 0.2 mV; GAD65-cre→n.m. 3/8, 3.6 ± 5.4 mV; VGLUT2→GAD65-GFP 9/15, 1.4 ± 1.6 mV; VGLUT2→n.m. 22/26, 2.8 ± 2.8 mV). Latencies from light to response onset were consistent with monosynaptic responses (ORX→GAD65-GFP 2.5 ms; ORX→n.m. 2.4 ms; GAD65-cre→ORX 11.5 and 8.2 ms; VGAT→ORX, 7.3 and 10.6 ms; ORX→n.m. 2.4 ms; MCH→n.m. 10.5 ± 5.0 ms; GAD65-cre→n.m. 5.6 ± 1.5 ms; VGLUT2→GAD65-GFP 7.6 ± 4.5 ms; VGLUT2→n.m. 6.4 ± 2.2 ms).

We wondered whether optogenetically evoked postsynaptic currents (oPSCs) would be similar to spontaneous synaptic currents (sPSCs), which typically arise from spontaneous discharge of single synaptic vesicles or individual presynaptic neurons. This comparison, which has never been documented, would offer an explanation wherein the previously demonstrated optogenetic connections may have arisen from an ultra-sparse connectivity coupled with activation of thousands of neurons, with possibly only one of them actually presynaptic to the recorded neuron. We therefore recorded oPSCs and sPSCs in continuous recordings within the same neurons (Figures 3D and 3E). Across cells, oPSCs were 61% bigger than sPSCs (sPSCs 20.3 ± 13.5 pA, oPSCs 32.8 ± 42.7 pA, p < 0.05; Figure 3F). However, the average oPSC amplitudes were typically within the distribution of sPSC amplitudes (Figure 3D). Furthermore, oPSCs were not significantly different (−37%, p > 0.05) from the average of the 5% largest sPSCs from each cell (top 5% sPSCs 52.8 ± 64.4 pA, oPSCs 32.8 ± 42.7 pA; Figure 3F), suggesting that the oPSCs can indeed arise from the firing of one connected presynaptic neuron. Rise time and decay time constants of oPSCs were similarly on a par with the top 5% sPSCs (Figure 3F). We should note these kinetic metrics may not be meaningful comparisons because the oPSCs were, more often than sPSCs, composed of compound multiphasic currents (e.g., biphasic rise in Figure 3D), which likely arise from occasional multiplet spikes in the presynaptic neuron (e.g., Figure 3A). In order to estimate how many presynaptic neurons might give rise to the oPSCs, we calculated oPSC/sPSC mean amplitude ratios for each cell (using the mean sPSCs rather than the top 5%). The average oPSC/sPSC ratio was 1.6 ± 0.9, and their distribution (Figure 3F) suggests that most oPSCs arise from 1 or 2 presynaptic neurons and even the largest oPSCs arise from 3–5 among thousands of activated neurons. This result is in line with our multiple patch-clamp recordings, and reconciles previous results with ultra-sparse connectivity within the LH.

### Lack of Locally Generated Oscillations in the LH

**Figure 4:**
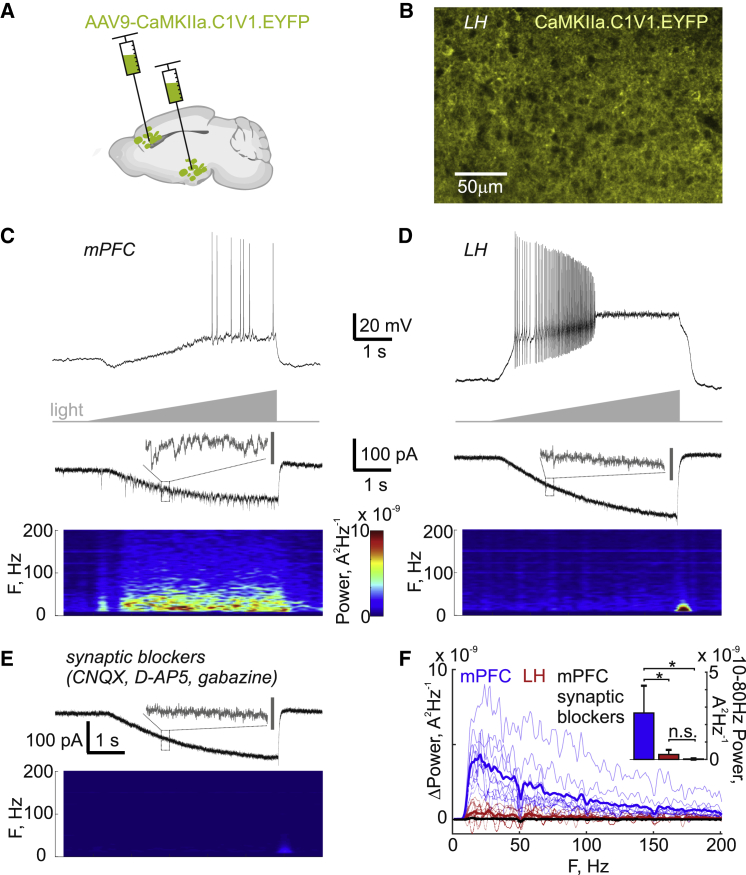
Optogenetically Induced Local Network Oscillations Are Absent in the LH (A) Schematic of opsin delivery to the LH and mPFC. (B) Example confocal micrograph of LH neurons showing abundant expression of CaMKII.C1V1.EYFP. (C) Example recordings in the mPFC. Top: example of light-ramp-induced firing in a current-clamp recording. Middle: voltage-clamp recording (−60 mV) during the light-ramp-induced synaptically driven oscillatory currents. Bottom: spectrogram showing increasing power in the beta-gamma band (10–80 Hz) during the light ramp. (D) Same as (C) but in the LH, showing the absence of beta-gamma oscillations. (E) Same voltage-clamp recording as in (C) after adding synaptic blockers, showing the oscillation is dependent on local synaptic connectivity. The color map in (C) applies to (D) and (E). The timescale is the same in all plots (C–E) except the gray insets in the voltage-clamp traces are expanded views of the indicated 200-ms segment (dashed box) and the gray scale bar is 50 pA (expanded views are scaled identically for comparison). (F) Power increase across frequency bands during the light ramp, showing the presence of light-driven beta-gamma oscillations in the mPFC (recordings from each cell in light blue, average in thick blue, n = 10) but not in the LH (recordings from each cell in light red, average in thick red, n = 10), and deletion of mPFC oscillations by synaptic blockade (recordings from each cell in gray, average in thick black, n = 5). Inset: average power in the 10- to 80-Hz band across cells. ^∗^p < 0.05; n.s., p > 0.05. Error bars denote SD.

Lastly, we asked what the functional effect of ultra-sparse connectivity is in the LH. A widely cited hypothesis on interregional communication postulates that efficient communication between brain regions is achieved through coherent oscillations, which relies on an intrinsic property for local circuits to oscillate [28]. Furthermore, the LH receives gamma-rhythmic input from the PFC and hippocampus, and a local gamma generator could be a basis for switching among these sources [29]. The dense excitatory and inhibitory connectivity within the neocortex allows it to generate local network oscillations in the 10- to 80-Hz beta-gamma band [30]. Does lack of dense connectivity in the LH mean that it cannot generate local network oscillations? We used the red-shifted opsin C1V1 driven by the ubiquitous neuronal CaMKII promoter, abundantly expressed in the mPFC and LH [31], to induce local network activity (Figures 4A and 4B). Because the dendrites of LH neurons are not consistently spatially organized, we were unable to measure local field potentials but instead had to use whole-cell recordings to measure rhythmic effects on transmembrane currents [22, 32]. We used linear light-intensity ramps over 5 s to drive network activity so that a possible “sweet spot” of network activation would not be missed. In current-clamp recordings, this depolarized all recorded neurons (10/10 in the LH and mPFC) and induced firing in 8/10 LH neurons and 4/10 in the mPFC (Figures 4C and 4D). However, we only found oscillations in the mPFC even though neuronal firing was reliably recruited in both the mPFC and LH (Figures 4C and 4D; n = 10 neurons in each area). To demonstrate that the membrane current oscillation in mPFC neurons was not due to intrinsic properties of the recorded neurons, which may differ from LH neurons, we washed on a cocktail of synaptic blockers that abolished the oscillation (Figures 4E and 4F; the average 10- to 80-Hz band power induced by the light ramp dropped by 99%, p = 10^−4^ by paired t test, n = 5). On average, LH neurons had 11% of the 10- to 80-Hz band-power increase seen in mPFC neurons during the light ramp, and the power in LH neurons was not significantly different from the power in mPFC neurons under synaptic blockade (Figure 4F). Therefore, these data suggest that the ultra-sparse connectivity in the LH makes it unable to generate local beta and gamma oscillations.

## Discussion

We found, with multi-neuron whole-cell recordings, that the LH does not contain neocortical-like densely connected microcircuits (Figures 1 and 2). Consistently, the few optogenetically identified connections within the LH were equivalent to spontaneously occurring PSCs (Figures 3D–3G). This suggests they arise from as few as one synaptic connection, which is remarkable given that thousands of neurons in the “presynaptic” population are likely to be activated. Because a quantitative comparison to previous work with similar and different optogenetic constructs is confounded by the lack of quantification of opsin spread and penetrance in the same brain slices as were used for electrophysiology, we limit our discussion to qualitative remarks. Although less comprehensive than ours, previous optogenetics studies also found predominantly single-synapse strength connections [15, 17, 18]. In contrast, optogenetically activated long-range sources of input to the LH are orders of magnitude stronger [33, 34]. A number of previous indirect electrophysiological and anatomical observations have shown the existence of peptidergic and synaptic connections within the LH [35, 36, 37, 38, 39, 40, 41, 42], but their frequency and strength are not clear. Therefore, they are compatible with our results, which for the first time establish the extreme sparseness and strength relative to sPSCs of the previously identified connectivity. We note that some indirect and optogenetic assays have not been benchmarked to distinguish between indirect responses from nearby neurons versus terminals of faraway neurons, whereas multi-neuron whole-cell recordings are.

The extreme sparseness of LH intraconnectivity has many implications. Because the LH does not contain a gamma generator (Figure 4) needed to receive and send gamma-rhythmic communications, an information transfer protocol based on gamma coherence [28, 43] is unlikely to be utilized by the LH. Gamma oscillations seen *in vivo* with local field potential recordings in the LH [44] could arise from the many passing axon bundles such as the fornix or incoming synapses [45]. Dense connectivity in the neocortex is based on inhibitory interneurons that migrate into the cortical scaffold from the ganglionic eminences during development [46]. The migrating interneurons do not go into the adjacent hypothalamus due to the presence of non-permissive factors that direct them into the frontal migratory streams toward the neocortex and striatum [47]. Thus, it may be that the hypothalamus lacks dense connectivity as a consequence of acting as a “sheepdog” to migrating interneurons. In line with this, two other subcortical circuits at the rostro-caudal level of the LH lack intrinsic synaptic connectivity, the subthalamic nucleus [14] and thalamic reticular nucleus [13]. It stands to reason that cortical microcircuits are more prone to circuit disorders such as epilepsy and schizophrenia, due to their recurrent excitatory local connectivity. Therefore, recurrent excitatory connectivity might pose a significant risk to survival in those vital hypothalamic networks that do not need local connectivity to achieve rhythmic output, such as the LH. Because nearby brain regions are thought to have arisen by duplication and elaboration in evolution [48], one intriguing possibility is that dense local connectivity could only evolve as an add-on at later stages in partially redundant structures. Lastly, the ultra-sparse connectivity in the LH implies that any integration and filtering of afferent input to the LH occur primarily within individual LH neurons. Consequently, coordination of activity in upstream networks is required for the rapid, coordinated activity of LH neurons during behavior [34, 49].

## STAR★Methods

### Key Resources Table

**Table:** 

REAGENT or RESOURCE	SOURCE	IDENTIFIER
**Bacterial and Virus Strains**		

AAV1-hORX.GCaMP6s (2.5 × 10^12^ GC/ml)	[49]	U Penn vector core N/A
AAV1-hORX.C1V1(t/s).mCherry (> 10^13^ GC/ml)	[49]	Vigene N/A
AAV2-EF1a.DIO.hChR2(E123T/T159C).mCherry (7.3 × 10^12^)	UNC GTC Vector Core	N/A
AAV9-CaMKIIa.C1V1(t/t).TS.EYFP (≥10^13^ CG/ml,)	Addgene	35499-AAV9

**Experimental Models: Organisms/Strains**		

Gt(ROSA)26Sor^tm14(CAG-tdTomato)Hze^	The Jackson Laboratory	007914
Slc17a6^tm2(cre)Lowl^	The Jackson Laboratory	016963
Slc32a1^tm2(cre)Lowl^	The Jackson Laboratory	016962
Gad2^tm2(cre)Zjh^	The Jackson Laboratory	010802
Pvalb^tm1(cre)Arbr^	The Jackson Laboratory	008069
Tg(Pmch-cre)1Lowl	The Jackson Laboratory	014099
Tg(Npy-hrGFP)1Lowl	The Jackson Laboratory	006417
GAD56-GFP	[50]	N/A

### Resource Availability

#### Lead Contact

Further information and requests for resources and reagents should be directed to the Lead Contact, Mahesh Karnani (mahesh.karnani@parisdescartes.fr).

#### Materials Availability

This study did not generate any unique reagents.

#### Data and Code Availability

Further information and requests for the datasets (please indicate requested file format) and code generated by this study should be directed to and will be fulfilled by the Lead Contact, Mahesh Karnani (mahesh.karnani@parisdescartes.fr). The data have not been deposited on a public repository due to file size and format limitations.

### Experimental Model and Subject Details

Animal handling and experimentation was approved by the UK government (Home Office) and by Institutional Animal Welfare Ethical Review Panel or carried out according to recommendations in the Animal Welfare Ordinance (TSchV 455.1) of the Swiss Federal Food Safety and Veterinary Office, and were approved by the Zürich Cantonal Veterinary Office. Animals of both sexes, aged 21-180 days at the beginning of the procedures were used and were housed in a controlled environment on a reversed 12h light-dark cycle with food and water *ad libitum*. Breeders were LSL-TdTom (Ai14), VGLUT2-cre (Slc17a6^tm2(cre)Lowl^), VGAT-cre (Slc32a1^tm2(cre)Lowl^), GAD65-cre (Gad2^tm2(cre)Zjh^), MCH-cre (Tg(Pmch-cre)1Lowl), GAD65-GFP [50] and WT C57BL6 mice for LH slices and NPY-GFP (Tg(Npy-hrGFP)1Lowl), PV-cre (Pvalb^tm1(cre)Arbr^) or VGAT-cre for mPFC slices, and were obtained originally from the Jackson Laboratory. Several strategies were used to obtain animals with two marker labeled populations in LH as follows. Crossing the breeders to yield VGLUT2-cre::LSL-TdTom::GAD65-GFP, MCH-cre::LSL-TdTom::GAD65-GFP, or VGAT-cre::LSL-TdTom, MCH-cre::LSL-TdTom, GAD65-cre::LSL-TdTom injected with an orexin promoter virus (ORX.GCaMP/ORX.C1V1) explained below, or a cre line injected with a mixture of a floxed virus and an orexin promoter virus explained below.

### Method Details

#### Virus injections

Mice were injected stereotactically with 100-150nl of AAV1-hORX.GCaMP6s (2.5 × 10^12^ GC/ml, U Penn vector core), AAV1-hORX.C1V1(t/s).mCherry (> 10^13^ GC/ml, Vigene), AAV2-EF1a.DIO.hChR2(E123T/T159C).mCherry (7.3 × 10^12^ UNC GTC Vector Core), or 300nl of 1:1 mixed AAV1-hORX.GCaMP6s and AAV2-EF1a.DIO.hChR2(E123T/T159C).mCherry, or 400nl of AAV9-CaMKIIa.C1V1(t/t).TS.EYFP (≥10^13^ CG/ml, Addgene). Orexin promoter virus expression specificity has been characterized previously [49, 51]. The used viral loads were similar to, or higher than, those used in compared studies [15, 16, 17, 18]. For surgery, mice were anesthetized with isoflurane, the scalp was infiltrated with lidocaine, opened, and a 0.2 mm craniotomy was drilled at 0.9 mm lateral, 1.4 mm posterior from Bregma. A pulled glass injection needle was used to inject virus 5.4 mm deep in the brain at a rate of 50 nl/min. The surgery was repeated similarly in the other hemisphere, except when using AAV9-CaMKIIa.C1V1(t/t).TS.EYFP, in which case the surgery was repeated in the other hemisphere at the PFC coordinates 0.4 mm lateral, 1.7 mm anterior from Bregma, and injection at 1.5 mm depth. After removal of the injection needle, the scalp was sutured and animals received 5 mg/kg carprofen injections for two days as post-operative pain medication. Virally injected animals expressed the proteins for an average of 48 ± 18.4 days before the experiment (range 27-91 days).

#### Preparation of acute slices

Coronal, sagittal or horizontal brain slices from P21-180 animals were prepared after instant cervical dislocation and decapitation. The brain was rapidly dissected and cooled in continuously gassed (95% O_2_ and 5% CO_2_), icy cutting solution containing (in mM): 90 N-methyl-D-glucamine, 20 HEPES, 110 HCl, 3 KCl, 10 MgCl_2_, 0.5 CaCl_2_, 1.1 NaH_2_PO_4_, 25 NaHCO_3_, 3 pyruvic acid, 10 ascorbic acid and 25 D-glucose. 350 μm thick coronal brain slices were cut on a vibratome (Campden) and allowed to recover for 5-15 min at 37°C in cutting solution, followed by 45-55 min at 22°C in artificial cerebrospinal fluid (ACSF) containing (in mM): 126 NaCl, 3 KCl, 2 MgSO_4_, 2 CaCl_2_, 1.1 NaH_2_PO_4_, 26 NaHCO_3_, 0.1 pyruvic acid, 0.5 L-glutamine, 0.4 ascorbic acid and 25 D-glucose, continuously gassed with 95% O_2_ and 5% CO_2_.

#### Slice electrophysiology

Patch clamp recordings were performed in a submerged chamber with 3-5 ml/min superfusion with ACSF, continuously gassed with 95% O_2_ and 5% CO_2_. A modified Olympus upright microscope with diascopic gradient contrast optics and episcopic fluorescence was used to identify neurons in slices. 3-7 MOhm patch pipettes were filled with intracellular solution containing (in mM): 130 K-gluconate, 5 NaCl, 2 MgSO_2_, 10 HEPES, 0.1 EGTA, 4 Mg-ATP, 0.4 Na-GTP, 2 pyruvic acid, 0.1 Alexa-594, 0.1% biocytin, and ∼10 mM KOH (to set pH to 7.3). Whole cell recordings were not analyzed if the access resistance was above 25 MOhm. Recordings were sampled at 10 or 20 kHz and low-pass filtered at 3 kHz with HEKA EPC10 usb amplifiers and acquired with HEKA patchmaster software. Current clamp data were recorded at by injecting a steady current to set membrane potential at −50mV. Action potential trains were elicited with 50Hz, 1ms, 1nA current steps. Voltage clamp data were recorded at a holding voltage of −60mV to study excitatory PSCs and 0mV to study inhibitory PSCs. These protocols and solutions have been used to reliably study cortical microcircuits in this paper (Figure 2) and others [20, 21, 22]. Electrical coupling coefficient was measured during a current step as the size of postsynaptic cell voltage change divided by size of presynaptic voltage change [27]. Opsins were stimulated with green (∼16 mW/mm^2^, for ORX-C1V1) or blue (∼10 mW/mm^2^, for ChR2) light from a xenon lamp (Sutter lambda 4DG controlled from HEKA patchmaster) through a TRITC-filter or with a 532 nm green laser (Laserglow) for linear light ramps from 0 to ∼20 mW/mm^2^. Patch clamp data were analyzed in MATLAB. Spectrograms were generated in MATLAB with the spectrogram function, using 0.5 s windows and 95% overlap, after high-pass filtering at 10Hz. Chemicals for making solutions were purchased from Sigma-Aldrich, except synaptic blockers CNQX (50μM), D-AP5 (50μM) and gabazine (3μM) which were from Tocris.

### Quantification and Statistical Analysis

All data are shown as mean ± s.d. unless stated otherwise. Statistical significance was determined by paired or unpaired Student’s t test or Wilcoxon signed rank test as stated. All statistics were performed using statistical functions in MATLAB.
